# Abscess of the cervical spine secondary to injection site infection in a heifer

**DOI:** 10.1186/s13028-017-0278-z

**Published:** 2017-01-31

**Authors:** Ueli Braun, Christian Gerspach, Karolin Kühn, Julia Bünter, Monika Hilbe

**Affiliations:** 1 Department of Farm Animals, Vetsuisse Faculty, University of Zurich, Winterthurerstrasse 260, 8057 Zurich, Switzerland; 2 Clinic of Diagnostic Imaging, Vetsuisse Faculty, University of Zurich, Winterthurerstrasse 260, 8057 Zurich, Switzerland; 3 Institute of Veterinary Pathology, Vetsuisse Faculty, University of Zurich, Winterthurerstrasse 260, 8057 Zurich, Switzerland

**Keywords:** Cattle, Neck, Injection, Abscess, Vertebral column, Spinal cord

## Abstract

**Background:**

Abscesses in the neck region can result from infection associated with injection of drugs into the neck muscles. To our knowledge, there have been no reports of osteomyelitis of the cervical vertebra and spinal cord compression secondary to an abscess in the neck. This case report describes the findings in a 9.5-month-old heifer with an abscess of the cervical spine secondary to injection site infection.

**Case presentation:**

The main clinical findings were swelling on the left side of the neck, proprioceptive deficits in all limbs and generalised ataxia. The ultrasonographic examination of the swelling showed an abscess. Radiographs showed a well-defined lytic lesion in 5th cervical vertebra (C5). Postmortem examination revealed an intramuscular encapsulated abscess on the left side of the neck at the level of C5. The abscess had invaded the vertebral canal and caused marked compression of the spinal cord.

**Conclusions:**

Injection technique is critical for the prevention of problems such as those described in this report. Sterile hypodermic needles must be used, and the volume of drug per injection site limited to 10–15 ml in young cattle.

## Background

Diseases of the cervical spine that involve the spinal cord occur occasionally in cattle. Some of the more common diseases include abscesses [1, 2], osteomyelitis [3] and discospondylitis [4–6], which result in generalised ataxia, proprioceptive deficits in the forelimbs and tetraparesis. Marked stiffness of the neck has also been reported in cattle with discospondylitis [4]. Other causes of cervical spine and spinal cord disorders include trauma, neoplasia, hypodermosis and degenerative lesions. Demeanour, mental status and cranial nerve function are normal in cattle with cervical spine and spinal cord lesions [2]. A tentative diagnosis of cervical spine disease is based on clinical findings and confirmed using radiography, computed tomography, magnetic resonance imaging and/or analysis of cerebrospinal fluid. Abscesses of the cervical spine usually result from haematogenous spread of infection. In a study of 14 calves with cervico-thoracic osteomyelitis, *Salmonella* Dublin was isolated from the vertebral lesions of eight calves [7]. Abscesses in the neck region caused by injection into the neck muscles or by using contaminated needles and resulting in cervical spinal cord damage and motor disturbances are briefly mentioned in two textbooks [8, 9]. However, a literature search in PubMed and VetMed Resource from 1975 to 2015 using the keywords *cattle*, *neck*, *abscess* and *injection* did not generate any results. This case report describes a dairy heifer with osteomyelitis of the 5th cervical vertebra (C5) and spinal cord compression secondary to an abscess in the neck.

## Case presentation

A 9.5-month-old Red Holstein heifer was referred in July to our clinic because of chronic weight loss and ataxia. Weight loss first was noticed 2 months before referral when she had been kept on an Alpine community pasture. During that time, the heifer had been examined by different veterinarians on several occasions and had received anthelmintics, penicillin/streptomycin, dexamethasone, vitamin ADE and selenium and had been vaccinated against ringworm even though ringworm lesions were present. Intramuscular injection of medications was done in the neck, shoulder and hind limb regions, but it was not possible to determine retrospectively the injection site for each drug. The heifer was in poor general health and obtunded, had a body condition score of 1.5/5 and weighed 185 kg (reference interval at 9.5 months, 180–295 kg). The rectal temperature was 38.9 °C, the heart and respiratory rates were 68 beats/min and 40 breaths/min, respectively and rumen motility and stratification were reduced. A 15 × 15 cm swelling was visible on the left side of the neck and passive bending of the head and neck was difficult. The left prescapular lymph node appeared normal on palpation. The heifer made minimal attempts to assume a normal stance after adduction, abduction and crossing of the fore- and hind limbs and had generalised ataxia. Extensor muscle tone was normal and there was no muscle atrophy. The extensor reflex was not evaluated. Other neurological findings including cranial nerve function were normal. The results of haematological examination including the leukocyte count (6700 leukocytes/µl, reference interval 5000–10,000 cells/µl), the activity of liver enzymes, the concentrations of bilirubin, urea and electrolytes and venous blood gas analysis were normal. Bovine viral diarrhoea virus antigen ELISA was negative.

**Fig. 1:**
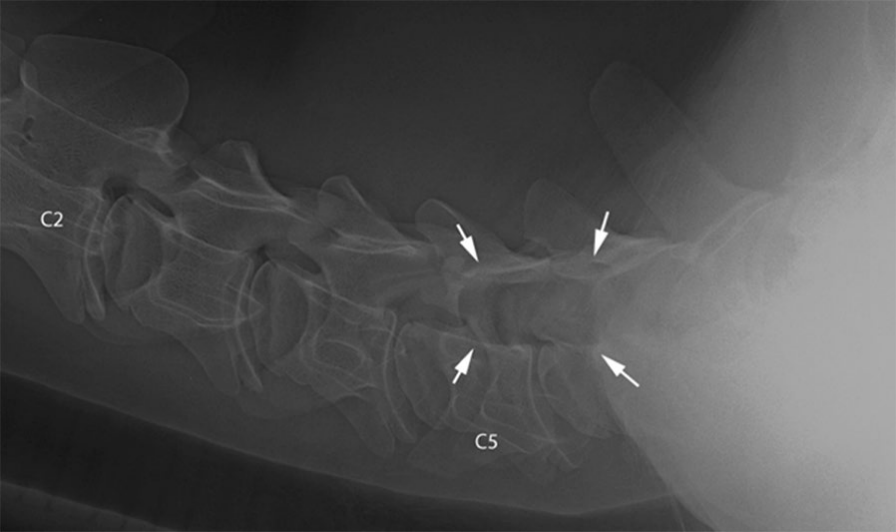
Laterolateral radiographic view of the cervical vertebral column. Laterolateral radiographic view of the cervical vertebral column of a 9.5-month-old Red Holstein heifer. The second (*C2*) and fifth (*C5*) vertebrae are labelled. A lytic lesion at the level of C5 and C6 (*arrows*) superimposed on the vertebral canal corresponds to the lateral parts of these vertebrae. The lesion has a thin sclerotic rim cranially

Ultrasound-guided collection of cerebrospinal fluid at the atlanto-occipital foramen [10] produced transparent colourless fluid with a protein content of 0.32 g/l and a leukocyte count of one cell/µl. Ultrasonographic examination of the neck swelling showed hyperechoic fluid with gaseous inclusions surrounded by a thin capsule indicative of an abscess in the subcutaneous tissues and neck musculature. Laterolateral, ventrodorsal and oblique radiographic views showed a soft tissue swelling on the left side of C4, C5 and C6 and a well-defined lytic lesion in C5, which involved the left half of the vertebral body, intervertebral lamina and transverse process (Fig. 1). Involvement of C6 was suspected even though there was poor delineation of the caudal part of the lesion because of superimposition of soft tissue swelling. The diagnosis based on radiographic findings was abscess of the left side of the neck with involvement of C5 and C6 and the spinal canal and possible spinal empyema. Faecal flotation showed gastrointestinal nematode (>1000 eggs/g) and *Trichuris* sp. (<200 eggs/g) eggs.

**Fig. 2:**
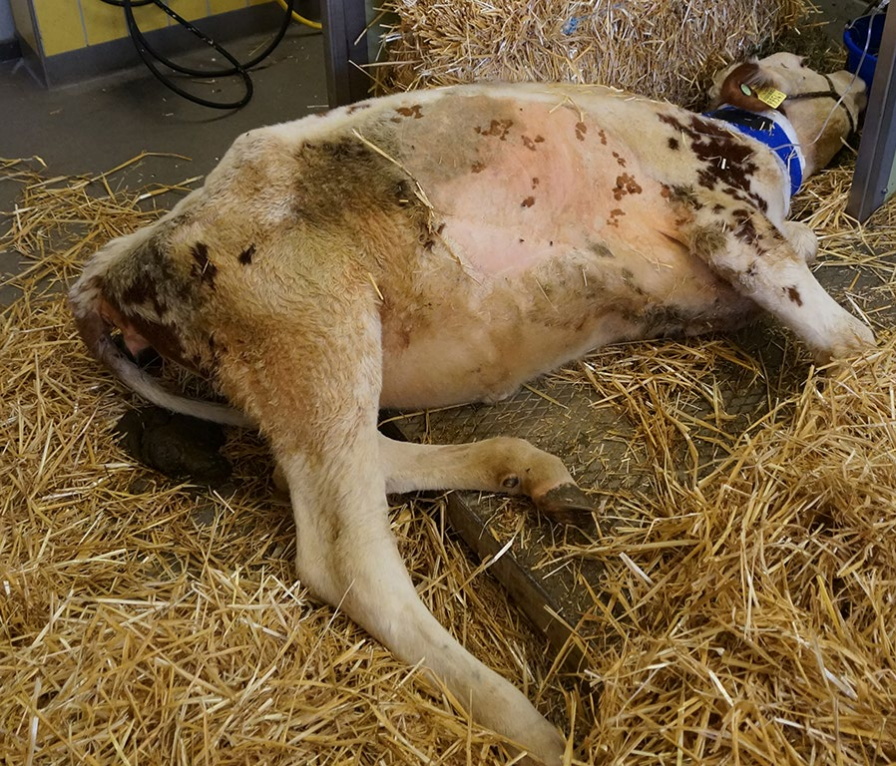
Heifer in lateral recumbency. A 9.5-month-old Red Holstein heifer in lateral recumbency and unable to rise because of a cervical spinal abscess secondary to an injection site infection in the neck

**Fig. 3:**
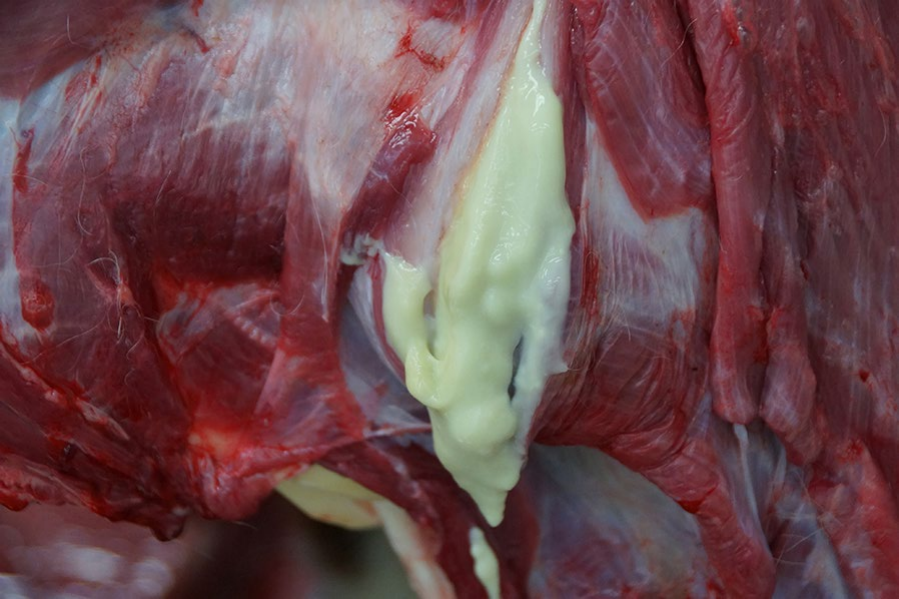
Postmortem view of incised abscess. Postmortem view of incised abscess in the neck muscle

**Fig. 4:**
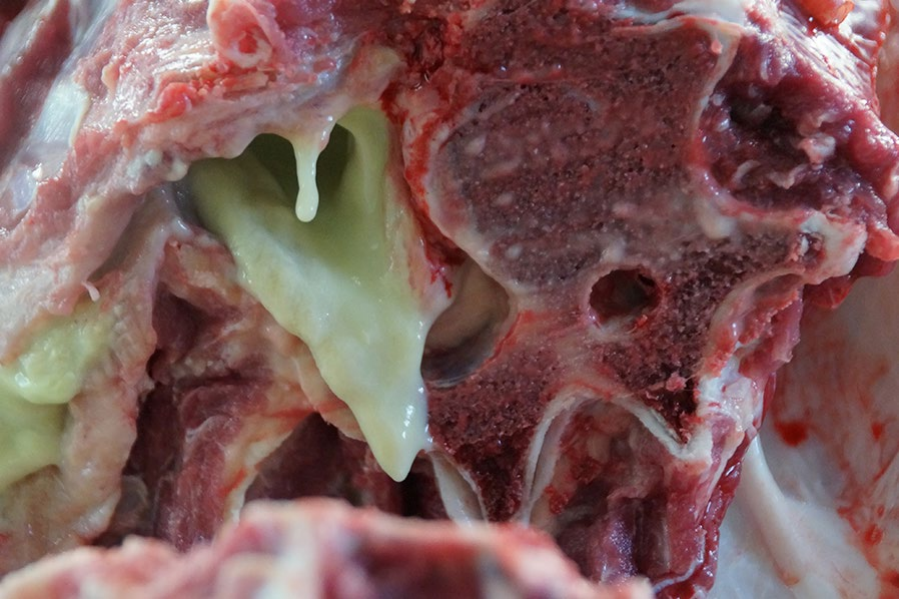
Postmortem view of the abscess extending into the vertebral column. Postmortem view of the abscess extending into the cervical vertebral column

**Fig. 5:**
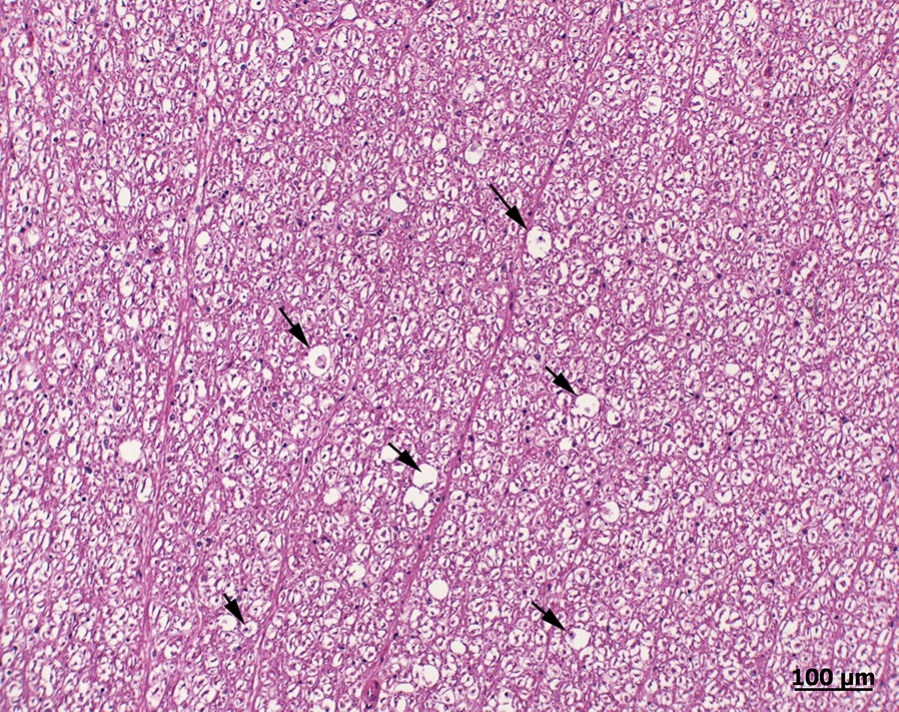
Histological findings in the *white* matter. Histological view of changes in the white matter of the compressed cervical spinal cord. Wallerian degeneration is characterised by multiple dilated myelin sheaths (*arrows*) containing myelinophages (*stars*)

Euthanasia of the heifer was elected because of progressive deterioration in condition and inability to stand (Fig. 2). Postmortem examination revealed an intramuscular encapsulated abscess on the left side of the neck at the level of C5. It contained yellow pus (Fig. 3) and extended to the cervical vertebral column (Fig. 4) and into the vertebral canal. There was severe damage to C5 and the left part of the vertebral arch was missing. The abscess had invaded the vertebral canal through this defect and caused marked compression of the spinal cord. Histological examination of the compressed spinal cord showed widespread axonal injury and myelin sheath dilation in the white matter of the spinal cord (Fig. 5). The cross-sectional appearance of the spinal cord including the normal butterfly shape of the grey matter was distorted.

The final diagnosis was severe apostematous myositis of the left neck region with extension of infection into the cervical vertebral column, resulting in severe focal osteomyelitis of the left part of the arch of C5 and marked compression of the spinal cord with moderate Wallerian degeneration. The heifer also had gastrointestinal parasitism. The poor condition of the heifer at initial presentation was thought to be due to parasitism and ringworm. No other diseases were detected at post-mortem examination.

Based on the results of the literature search, this is the only published case of injection site abscess with involvement of the vertebral column in cattle. However, it can be assumed that iatrogenic lesions of the neck musculature accompanied by abscess formation with involvement of the cervical vertebral column are common. Contaminated hypodermic needles can result in abscess formation [9], and several antibiotics, sulfonamides and vitamin preparations are known to cause severe muscle reactions, particularly after repeated injections [8]. These muscle changes may progress to connective tissue induration, necrosis or abscess formation, and resulting cervical spinal cord damage accompanied by motor deficits has been described in a textbook [8]. Epidural spinal abscess usually is the result of vertebral osteomyelitis caused by *Trueperella pyogenes* or *Fusobacterium necrophorum* [9]. Other authors also consider the haematogenous spread of *Trueperella pyogenes* a common aetiology of vertebral and epidural abscesses [11]. The cause of cervical vertebral abscesses in cattle could not be definitively determined in one report [2] and in another, haematogenous spread of infection was suspected because four of five affected cattle also had abscesses in the lungs or thorax [1].

The absence of changes in cerebrospinal fluid was thought to be due to the extramedullary location of the abscess, which did not affect the integrity of the dura mater. In fact, the dura mater is almost always impermeable to infectious processes [9]. Pachymeningitis is associated with a markedly increased leukocyte count and protein concentration in the cerebrospinal fluid [12]. The histological findings were attributable to compression of the spinal cord by the abscess.

Progressive paresis and paralysis seen in the present case were typical signs of a space-occupying lesion in the vertebral canal [11]. Early signs include difficulty rising followed by ataxia, weakness, dragging of the toes and knuckling as the spinal cord compression worsens [11]. In the final stages, affected cattle require assistance when rising and eventually become recumbent. Paresis may occur suddenly when osteomyelitis leads to fracture of a vertebral body and bone fragments enter the vertebral canal and compress or injure the spinal cord. The clinical signs were typical of an upper motor neuron lesion [13], which is characterised by loss of voluntary movement, impaired proprioception, normal or increased muscle tone, mild muscle atrophy caused by disuse in chronic cases and normoreflexia or hyperreflexia.

## Conclusion

Injection technique is critical for the prevention of problems such as those described in this report. Subcutaneous injection of drugs is the preferred route in calves and intramuscular injection of drugs that also are labelled for subcutaneous should be avoided. However, when intramuscular injection is required in dairy calves, the posterior thigh muscles should be used. Sterile hypodermic needles must be used, and the volume of drug per injection site limited to 10–15 ml in young cattle.
